# A novel approach of high speed scratching on silicon wafers at nanoscale depths of cut

**DOI:** 10.1038/srep16395

**Published:** 2015-11-09

**Authors:** Zhenyu Zhang, Dongming Guo, Bo Wang, Renke Kang, Bi Zhang

**Affiliations:** 1 Key Laboratory for Precision and Non-Traditional Machining Technology of Ministry of Education, Dalian University of Technology, Dalian 116024, China; 2 Changzhou Institute of Dalian University of Technology, Changzhou 213164, China; 3 State Key Laboratory of Metastable Materials Science and Technology, Yanshan University, Qinhuangdao 066004, China; 4 Department of Mechanical Engineering, University of Connecticut, Storrs, CT 06269, USA

## Abstract

In this study, a novel approach of high speed scratching is carried out on silicon (Si) wafers at nanoscale depths of cut to investigate the fundamental mechanisms in wafering of solar cells. The scratching is conducted on a Si wafer of 150 mm diameter with an ultraprecision grinder at a speed of 8.4 to 15 m/s. Single-point diamonds of a tip radius of 174, 324, and 786 nm, respectively, are used in the study. The study finds that at the onset of chip formation, an amorphous layer is formed at the topmost of the residual scratch, followed by the pristine crystalline lattice beneath. This is different from the previous findings in low speed scratching and high speed grinding, in which there is an amorphous layer at the top and a damaged layer underneath. The final width and depth of the residual scratch at the onset of chip formation measured vary from 288 to 316 nm, and from 49 to 62 nm, respectively. High pressure phases are absent from the scratch at the onset of either chip or crack formation.

Solar energy is extremely abundant. The amount of solar energy that hits the earth in merely 40 min can support the global energy consumption in one year. With the rise of carbon dioxide levels, renewable energy sources receive more attentions^1^,^2^,^3^,^4^. This makes the annual growth rate of photovoltaic (PV) industry over 40% during the past decade^5^. About 80% of solar cells in the PV industry are fabricated using crystalline silicon (Si)^6^,^7^,^8^,^9^. At present, multi-wire sawing is the most commonly used to cut wafers from an ingot in the PV industry^10^. Sawing cost occupies about 30% for wafering, and up to 50% amount of Si material is lost as kerf during multi-wire sawing^11^,^12^. Under the pressure of solar cell cost, the thickness of Si wafers used for PV solar cells decreases to about 150 μm^13^, and the abrasives employed in multi-wire sawing turns into smaller and smaller to reduce both the kerf loss and thickness of a defect layer left on Si wafers after sawing. Recombination of the minority carriers induced by defect layer significantly reduces the energy conversion efficiency of Si solar cells^14^. Up to date, monocrystalline Si continues to provide the highest energy conversion efficiency in all commercial PV modules^5^. This is because of the ultralow defects in monocrystalline Si solar cells, compared to multicrystalline and amorphous ones. Defects consist of amorphous phase, dislocation and cracks, which affect significantly the performance and reliability during handling and processing solar cells^15^, especially for thin PV solar cells with thickness less than 150 μm^13^,^16^. Fundamental mechanisms involved in wafering of solar cells are essential in solar cell fabrication, to reduce the breakage and warpage rates for thin PV solar cells.

Multi-wire sawing used in PV industry consists of numerous abrasives, and defects induced by a single abrasive involved in multi-wire sawing form fundamental issue in solar cell fabrication. The abrasives used in wafering of solar cells become smaller and smaller, to reduce the kerf loss and thickness of defect layer on Si wafers. For instance, abrasives with sizes ranging from 0.5 to 6 μm are used to lap and polish the cross-sections of film solar cells classified as next-generation ones^17^. The cutting speed of multi-wire sawing varies from 10 to 15 m/s^18^,^10^. This is extremely difficult with a single-point diamond tip of a tip radius at the sub-micron level, due to the absence of an experimental approach.

Single-point diamond tip scratching on Si wafers is fundamental to explore the mechanism involved in wafering of solar cells, thus attracting attentions^17^,^19^,^20^,^21^. Three typical approaches for single-point diamond tip scratching consist of instrumented nanoscratching^17^, atomic force microscopy (AFM) scratching^19^, and precision motion stage scratching^20^. The instrumented nanoscratching is performed using a commercial TI900 TriboIndenter (Hysitron, USA). AFM scratching is carried out by a commercial AutoProbe M5 (Park, USA). Precision motion state scratching is conducted on ANT-4 V (Aerotech, USA). The scratching speeds used in instrumented nanoscratching, AFM scratching, and precision motion stage scratching are 0.1–1 μm/s, 5 μm/s, and 17 μm/s, respectively, and their diamond tip radii are 100 nm, 100 nm, and 5–10 μm, correspondingly. Scratching speeds available in three approaches for single-point diamond tip scratching are 6 to 8 orders magnitude lower than that of abrasives used in multi-wire sawing (10–15 m/s)^18^,^10^ for solar cell fabrication. Additionally, the diamond tips used in the three approaches have their radii from 100 nm to 5 μm, leaving a huge gap in between. Presently, it is extremely difficult to perform pragmatic sawing at speeds of 10–15 m/s with the three approaches. It is a challenge to develop a novel approach to conduct high speed m/s scratching at the nanoscale depth of cut using a single-point diamond tip. This is attributed to the difficulties in fabricating a diamond tip with a sub-micron radius, carrying out m/s scratching at the nanoscale depth of cut, and identifying the onset of chip or crack formation. Nevertheless, it is intriguing to develop a novel approach to conduct high speed scratching, to elucidate the fundamental mechanisms involved in wafering of solar cells.

In this study, a novel approach of high speed scratching at the nanoscale depth of cut is developed, using three single-point diamond tips with a sub-micron radius. The fundamental mechanisms involved in wafering of solar cells are investigated at the onsets of chip and crack formation.

## Results

**Figure 1:**
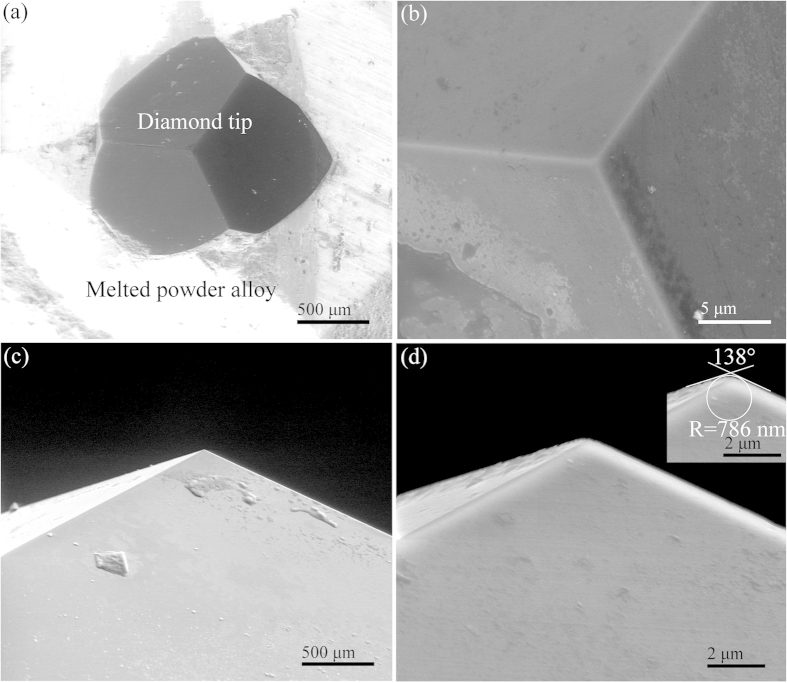
SEM images of top (**a,b**) and side (**c,d**) views at low (**a,c**) and high (**b,d**) magnifications of diamond tip B after scratching at speed of 15 m/s.

Figure 1 shows the SEM images of top and side views of diamond tip B at low and high magnifications after scratching at speed of 15 m/s. After high speed scratching, diamond tip B remained unchanged in its profile with no observable wear tracks. Three facets meet at one point, as shown in Fig. 1(b), showing the sharpness of the diamond tip. This verifies the validity of the diamond tips after high speed scratching at the nanoscale depth of cut. The radius of diamond tip B was 786 nm (Fig. 1(d)), consistent with the sizes ranging from 0.5 to 6 μm of the diamond grits used for fabrication of film solar cells^17^. Therefore, diamond tip B was of a typical size among the three single-point diamond tips prepared for the study.

**Figure 2:**
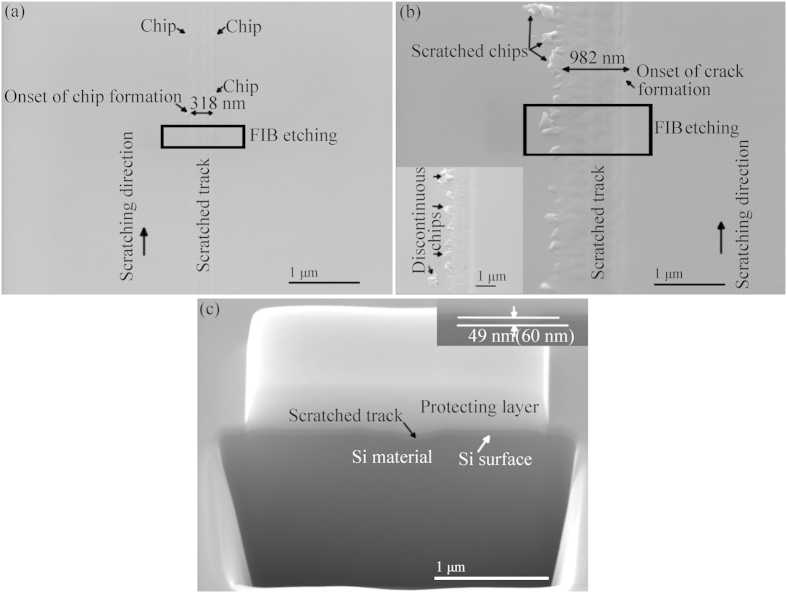
SEM images at the onset of (**a**) chip and (**b**) crack formations induced by diamond tip B, and (**c**) *in situ* FIB etching of the area marked with a black square in (**b**). Inset in (**b**) shows the discontinuous chips neighboring to the onset of crack formation. Inset in (**c**) shows the final depth of residual scratch at the onset of crack formation measured by SEM and actual depth etched by FIB listed in a bracket.

**Figure 3:**
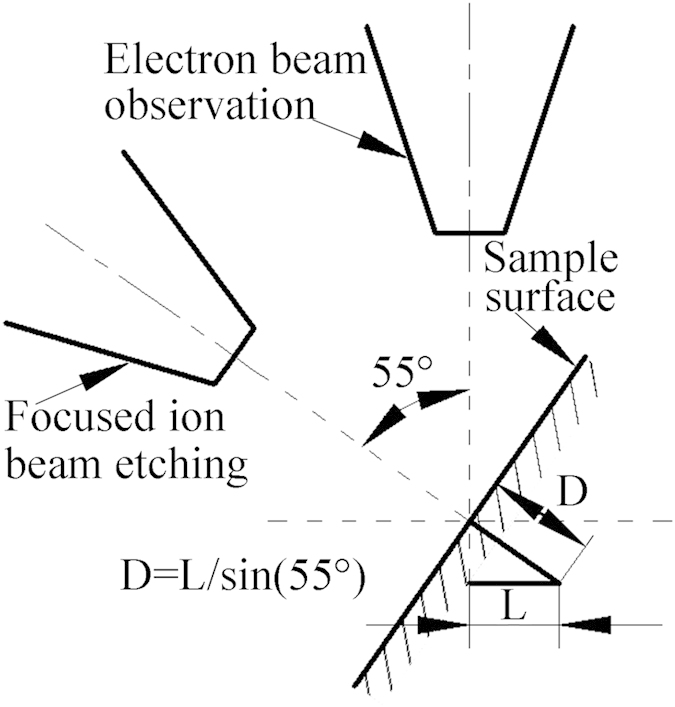
Schematic diagram of relationship between electron beam observation and FIB etching.

Figure 2 shows the SEM images at the onset of chip and crack formations and *in situ* FIB etching marked with a black square in (b). The widths at the onset of chip and crack formations are 318 (Fig. 2(a)) and 982 nm (Fig. 2(b)), respectively. The onset of chip and crack formations can be identified from the SEM images shown in Fig. 2. Discontinuous chips are observed in the inset of Fig. 2(b), which is in a good agreement with the previous reports performed at low speed scratching^20^. The final depth of the residual scratch at the onset of crack formation measured by SEM was 49 nm, as shown in the inset of Fig. 2(d), and the actual depth etched by FIB was 60 nm. A schematic diagram of relationship between electron beam observation and FIB etching is illustrated in Fig. 3. FIB etching was perpendicular to the sample surface, and electron beam observation, i.e. SEM measurement, was taken at a tilting angle of 45° from the sample surface. The relationship between the length measured by SEM, L, and the actual depth etched by FIB, D, is expressed,


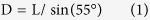


The widths at the onset of chip formation induced by the three diamond tips varied from 288.2 and 316.4 nm, and depths at the onset of crack formation changed from 48.7 to 62.1 nm, as listed in Table 1.

**Figure 4:**
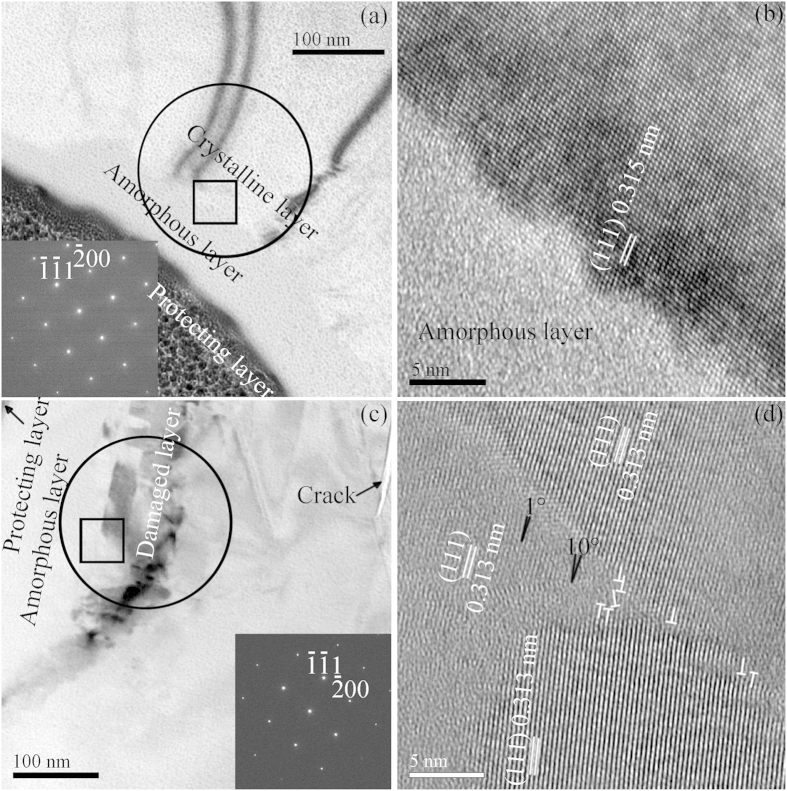
Cross-sectional TEM images at the onset of (**a,b**) chip and (**c,d**) crack formations formed by diamond tip B. (**b**) and (**d**) are the magnified area marked in the black squares of (**a**) and (**c**), respectively. Insets in (**a**) and (**c**) showing the corresponding SAED patterns taken from the black circles.

Figure 4 depicts cross-sectional TEM images at the onset of chip and crack formations formed by diamond tip B. At the onset of chip formation, an amorphous layer was observed at the topmost, followed by the pristine crystalline lattice underneath, which are confirmed by perfect diamond cubic Si-I phase of Si (111) plane in Fig. 4(a,b). This finding is different from that reported in previous literature^22^,^23^,^24^, in which an amorphous layer at the top is followed by a damaged layer beneath, generated by the low speed scratching^22^ and the high speed grinding using ultrafine diamond grits^23^,^24^. A crack is observed in Fig. 4(c), and grains with [111] orientations with their respective rotation angles of 1° and 10° are found in Fig. 4(d). At the onset of crack formation, there is an amorphous layer at the top and a damaged layer at the bottom, observed in Fig. 4(c,d). This agrees well with the previous findings^22^,^23^,^24^. In addition, at the onset of chip and crack formations, Si-I phase is identified using selected area electron diffraction (SAED) patterns in the insets of Fig. 4(a,c). This is distinct from the previous reports^22^,^23^ which claim that high pressure Si-III and Si-XII phases are present, and are produced by low speed scratching^22^ and multi-pass high speed nanogrinding^23^. Thus, in this study, high pressure phases of Si are absent from the scratched Si sample, at the onset of chip and crack formations.

## Discussion

**Figure 5:**
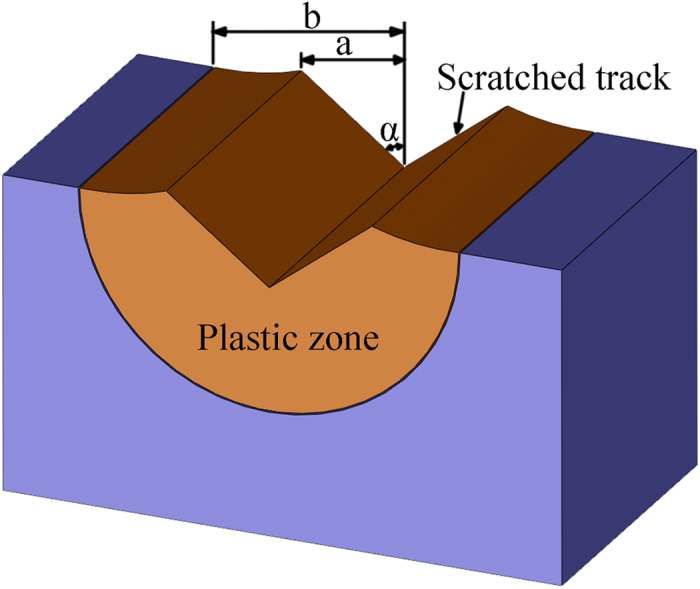
Schematic of plastic zone induced by a diamond tip.

To understand the underlying mechanisms of the onset of chip and crack formations, it is necessary to analyze stress and displacement of chip and crack formations. Figure 5 shows the schematic of plastic zone induced by a diamond tip. In Fig. 5, a, is the half width of the residual scratch, and α is the half included angle of a diamond tip listed in Table 1. The half size of plastic zone, b, is calculated^25^:


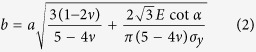


where *v* is Poisson’s ratio, *E* is Young’s modulus, and *σ*_*y*_ is yield strength. For Si (111) plane, *v* and *E* are 0.3 and 169.2 GPa, respectively^26^. *σ*_*y*_ is obtained^19^:


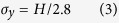


where *H* is hardness, and equal to 14.5 GPa for Si (111) plane^26^.

The normal force, *F*_*n*_ is addressed^25^:


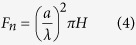


where λ is a dimensionless constant, taking 1.25 for asymmetric diamond tips^25^.

Lateral force, *F*_*l*_ is presented:


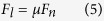


where *μ* is friction coefficient, taking 0.12 for Si (111) plane^27^.

**Figure 6:**
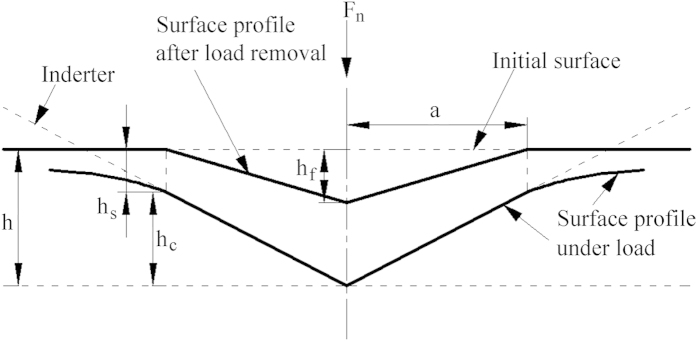
Schematic of cross-sectional surface profile under and after load applied by a diamond tip.

The effective indentation modulus, E* is given^28^:


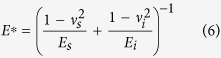


where *E* and *v* are the Young’s moduli and Poisson’s ratios of the sample (s) and indenter (i). For diamond tip, *E*_*i*_ and *v*_*i*_ are 1141 GPa and 0.07, respectively^28^. The total displacement *h* is consistent with Hertzian elastic contact^29^:


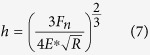


where *R* is the tip radius of the diamond grit. Figure 6 depicts the schematic of cross-sectional surface profile under and after load applied by a diamond tip. The displacement of the surface at the perimeter of the contact, *h*_*s*_ is determined^28^:


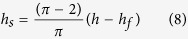


where *h*_*f*_ is the final depth of residual scratch after unloading, corresponding to the depth at the onset of crack formation listed in Table 1. In Fig. 6, the contact depth *h*_*c*_ is written:


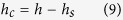


The stress σ is computed^28^:


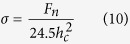


Table 2 shows the calculated forces, sizes and stress at the onset of chip and crack formations. At the onset of chip formation, the normal force applied on three diamond tips varies from 605 to 729 μN, which is very subtle and less than 1 mN. This results in the total displacement of the three diamond tips changing from 25 to 38 nm. The final depth of a residual scratch is much smaller than the total displacement, which is difficult to identify by the electron beam measurement. As the scratching speed of three diamond tips varies from 8.4 to 15 m/s, and the normal force is subtle, the deformation induced in the Si sample by a diamond tip is limited in a shallow surface. In addition, the Si sample has the diamond cubic structure of Si-I phase, which is difficult to deform. Under stress, the diamond cubic Si-I phase usually transforms to amorphous phase^22^,^23^,^24^. Subjected to subtle normal force and high speed scratching, the Si sample is formed with an amorphous layer at the topmost, followed by the pristine crystalline lattice, as shown in Fig. 4(a,b). This is distinct from the low speed scratching and multi-pass high speed nanogrinding, where high pressure phases and nanocrystals are present^22^,^23^. Under the influence of the normal force, the amorphous phase of the Si sample recrystallizes and transforms, producing nanocrystals and high pressure phases^30^. It interprets that nanocrystals and high pressure phases coexist in the amorphous phase in low speed scratching and high speed nanogrinding. Pure Si wafers oxidize in air, creating a layer of amorphous silica with a thickness of several and tens of nanometers. Thereby, high speed scratching actually occurs in the amorphous silica layer, rather than the pure Si. Prior to the onset of chip formation, ploughing takes place, without chips generated. Cutting happens after the onset of chip formation. For this reason, multi-wire sawing used for solar cells conducts on amorphous silica, generating and removing amorphous Si, rather than crystalline Si. Due to the intimate contact between the Si crystal and saw wire in solar cell fabrication, the subsequent polishing and etching is always on amorphous Si. Under the pressure of production cost in the PV industry, Si wafers are made thinner and thinner, and abrasives used in sawing for solar cells become smaller and smaller. On this account, the next-generation technology developed for wafering of solar cells aims to produce thinner and thinner amorphous Si to save Si materials and cost. The primary objective of polishing and etching after sawing applied in solar cells is to remove the amorphous Si.

At the onset of crack formation, the normal force exerted on the three diamond tips varies from 2472 to 8786 μN, corresponding to the total displacement changing from 92 to 174 nm, increasing about one order of magnitude compared to that at the onset of chip formation. This determined the final depth of the residual scratch. The normal stress loaded on the three diamond tips varies from 17.4 to 32.8 GPa, bringing about cracks taking place on the Si surface (Fig. 4(b)) and in the subsurface (Fig. 4(c)). However, the normal force at the onset of crack formation is relatively large, resulting in the release of stress downward and forming a damaged layer beneath the amorphous layer (Fig. 4(c)). Additionally, the high stress induced on the free surface produces cracks (Fig. 2(b)).

In summary, a novel approach of fabricating diamond tips from natural diamond grits is developed through grinding with diamond wheels and chemical finishing on a low carbon steel plate. The radius of the diamond tips is at the sub-micron level, and the included angle is varied from 135 to 140°. Si (111) wafers are used in the study. High speed m/s scratching at the nanoscale depth of cut is performed on an ultraprecision grinder with the spindle-face runout of 50 nm cooperating with flatness of 100 nm on the Si wafer. At the onset of chip formation, an amorphous layer is formed on the pristine crystalline lattice, without a damaged layer. This is distinct from the previous findings in which there is a damaged layer beneath the amorphous layer.

## Methods

### Development of three diamond tips from natural diamond grits

**Figure 7:**
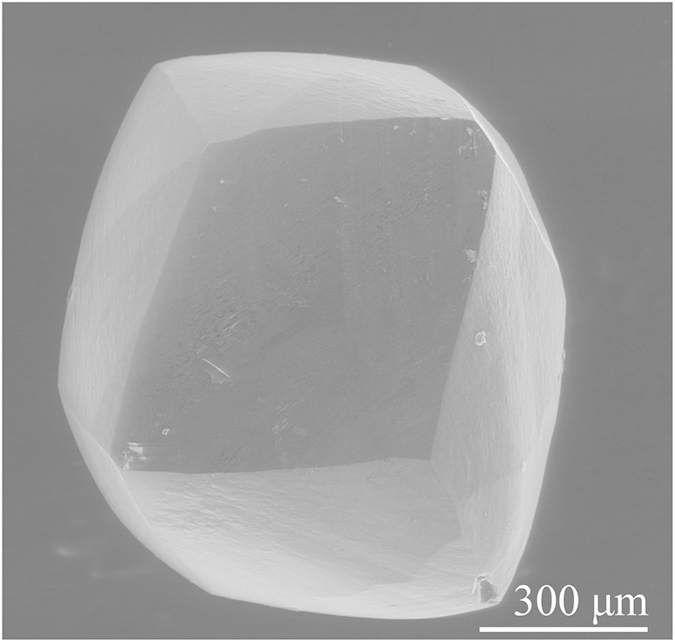
SEM image of a diamond grit.

Because of their impact strength in high speed scratching, natural diamond grits have been used for single-point diamond tips. The natural diamond grits are mostly produced in South Africa with a typical weight of 0.1–0.2 carats. Figure 7 shows the scanning electron microscopy (SEM) image of a diamond grit, which is 1 mm in length and 0.9 mm in width. Based on the texture of natural diamond grits, the hardest face was identified and marked in this study. A hole was drilled in one end of a carbon steel lever to hold the diamond grit. Size of the hole was two times as that of the diamond grit. The gap between the diamond grit and carbon steel lever was filled with nickel-based alloy powders. High-frequency melting was applied to fixing the diamond grit in the carbon steel lever, in which a graphite rod drilled with a hole in a size similar to the diamond grit was used to press the diamond grit. The marked face of the diamond grit was always upright, keeping the hardest face as the diamond tip. After the high-frequency melting, the diamond grit was ground using diamond wheels with sizes of 40, 20, 5, and 2 μm in sequence. Three-faceted pyramidal (3FP) diamond tips were fabricated using a dividing apparatus. Finally, a low carbon steel plate was used to finish the 3FP diamond tips, eliminating the damaged layer induced by diamond grinding. This is because of the chemical diffusion of diamond carbon atoms to the low carbon steel at high temperature during the grinding process. The three 3FP diamond tips developed are listed in Table 1. The diamond tips were designated as A, B, and C with their tip radii of 174, 786, 324 nm, respectively, and included angles of 140, 138, 135°, correspondingly. The radii of the three diamond tips were at the sub-micron level, filling the gap between nanometer and micrometer radii of the diamond tips used for the current μm/s low speed scratching. Their included angles were varied from 135 to 140°, close to that of the commercial Berkovich diamond tip (142.35°)^21^. It is noted that diamond tip A of tip radius of 174 nm and included angle of 140° is similar to the commercial Berkovich diamond tip, whose tip radius ranging from 150 to 200 nm and included angle being 142.35° 21.

### High speed m/s scratching at nanoscale depth of cut

**Figure 8:**
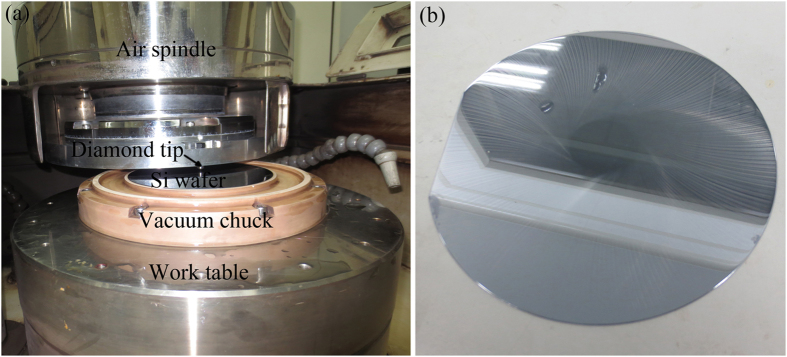
Optical images of (**a**) diamond tip fixed on an ultraprecision grinder, and (**b**) Si wafer subjected to high speed scratching.

To verify the validity of the three diamond tips developed at high speed m/s scratching, a diamond tip was mounted on an ultraprecision grinder (Okamoto, VG401 MKII, Japan), as shown in Fig 8. Si (111) wafer of 150 mm in diameter was used as specimen. High speed scratching was performed at a nanoscale depth of cut between the air spindle face of the ultraprecision grinder (50 nm runout) and the Si wafer of 100 nm flatness. Firstly, the air-spindle was fed downward manually until a touchdown of the diamond tip with the Si wafer. This is confirmed by a subtle scratch of the diamond tip on the Si wafer. A digital readout was taken upon the touch down, and then the air spindle was uplifted by 15 μm. Secondly, the diamond tip was rotated and fed downward at a speed of 1 μm/min, to the same digital readout previously taken, ceasing the rotation of the air-spindle and uplifting it instantaneously. Finally, the air-spindle was uplifted rapidly, finishing the high speed scratching process. Since the cutting speed of the multi-wire sawing employed in solar cells varies from 10 to 15 m/s^10^,^4^, the scratching speed of three diamond tips changed from 8.4 to 15 m/s, as listed in Table 1. A Si wafer after high speed scratching at a nanometer depth of cut is shown in Fig. 2(b). Scratches on the Si wafer were subtle and separated.

### Characterization

The diamond tips were characterized prior to and after high speed scratching by SEM (Lyra3 Tescan, Czech Republic). The SEM equipped with focused ion beam (FIB) was also used to characterize the scratches induced by the three diamond tips. The observation using the SEM was conducted from the initial touch down along the scratch to identify the onset of chip and crack formations, followed by an *in-situ* FIB etching to measure the depth of scratches and to prepare transmission electron microscopy (TEM) samples. TEM observations were conducted using an FEI Tecnai F20 microscope operated at an accelerated voltage of 200 kV.

## Additional Information

**How to cite this article**: Zhang, Z. *et al.* A novel approach of high speed scratching on silicon wafers at nanoscale depths of cut. *Sci. Rep.* **5**, 16395; doi: 10.1038/srep16395 (2015).

## Figures and Tables

**Table 1:** Nanoscratching conditions performed by developed three-faceted pyramidal single diamond tips.

Diamond tip	Included angle (degree)	Tip radius (nm)	Width at the onset of chip formation (nm)	Onset of crack formation		Wheel speed (m/s)	Table speed (rpm)	Feed rate of wheel (μm/min)
				Width (nm)	Depth (nm)			
A	140	174	303.5 ± 12.5	582.5 ± 16.4	48.7 ± 7.3	15	80	1
B	138	786	316.4 ± 10.6	984.5 ± 17.9	62.1 ± 6.7	15	80	1
C	135	324	288.2 ± 12.2	1098.2 ± 34.5	51.2 ± 8.1	8.4	80	1

**Table 2:** Calculated forces, sizes and stress at the onset of chip and crack formations.

Diamond tip	Onset of chip formation				Onset of crack formation					
	*F*_*n*_ (μN)	*F*_*l*_ (μN)	*b* (nm)	*h* (nm)	*F*_*n*_ (μN)	*F*_*l*_ (μN)	*b* (nm)	*h* (nm)	*h*_*c*_ (nm)	σ (GPa)
A	671	81	295	38	2472	297	566	92	76	17.4
B	729	88	315	25	7061	847	980	112	94	32.8
C	605	73	297	29	8786	1054	1132	174	129	21.5
